# Upper arm circumference development in Chinese children and adolescents: a pooled analysis

**DOI:** 10.1186/s40101-015-0062-6

**Published:** 2015-05-30

**Authors:** Fang Tong, Tong Fu

**Affiliations:** The Capital Institute of Pediatrics, Room 415, Science Building, No.2 Ya Bao Road, Beijing, 100020 People’s Republic of China; Beijing Information Science and Technology University, Beijing, China

**Keywords:** Upper arm circumference, Chinese children and adolescents, Pooled analysis

## Abstract

**Background:**

Upper arm development in children is different in different ethnic groups. There have been few reports on upper arm circumference (UAC) at different stages of development in children and adolescents in China. The purpose of this study was to provide a reference for growth with weighted assessment of the overall level of development.

**Methods:**

Using a pooled analysis, an authoritative journal database search and reports of UAC, we created a new database on developmental measures in children. In conducting a weighted analysis, we compared reference values for 0~60 months of development according to the World Health Organization (WHO) statistics considering gender and nationality and used *Z* values as interval values for the second sampling to obtain an exponential smooth curve to analyze the mean, standard deviation, and sites of attachment.

**Results:**

Ten articles were included in the pooled analysis, and these articles included participants from different areas of China. The point of intersection with the WHO curve was 3.5 years with higher values at earlier ages and lower values at older ages. Boys curve was steeper after puberty. The curves in the studies had a merged line compatible. The Z values of exponential smoothing showed the curves were similar for body weight and had a right normal distribution.

**Conclusions:**

The integrated index of UAC in Chinese children and adolescents indicated slightly variations with regions. Exponential curve smoothing was suitable for assessment at different developmental stages.

## Introduction

Upper arm circumference (UAC) assessment reflects comprehensive growth in children, such as growth of skeleton, muscles, and subcutaneous fat, which has been used as an alternative indicator of nutritional status if collection of height and weight measurements was difficult. Previous Chinese reports have concentrated on assessment during the 2000s, and these reports mainly focused on malnutrition in children [1].

There are some differences in UAC among different racial or ethnic groups: the general population and athletes or people who do physical labor for a living. Adult muscle strength in the African, European, and Asian populations are different, with Asians being slightly inferior in terms of UAC. There are also some differences among ethnic groups. According to the anthropological human body physique study performed by the Chinese National Natural Foundation [2–5], clustering analysis showed that the male Han Chinese urban and rural area populations in Jiangsu, Uzbeks, Russians, Kazaks, and 10 other northern ethnic groups had a UAC that was about 28 cm, which would be considered as medium dimensions. The average mean of other groups were 30 to 24 cm for the highest to lowest UAC. Women had a lower average about 2 cm both medial and other groups.

Although there are these differences in adults, as well as the uncertainty of muscle development rates in youngsters [6], WHO study was revised UAC by age and gender from 0 to 60 months (http://www.who.int/childgrowth). A pooled analysis of populations in Norway, Brazil, Hong Kong, and a total of seven other nationalities was carried out based on growth. Little obvious differences during the period that was studied were found, and this study is considered the representative reference.

National data on children’s growth and development was also published by the Chinese Public Health Ministry, and the data were mainly about height, weight, and head circumference by age and gender, but the study did not clarify UAC. Searching the published data to date, articles have mainly described results of region’s survey. It is therefore necessary to carry out a pooled analysis to determine the UAC growth conditions of children and adolescents in Chinese population, which is one fourth of the world’s population.

## Methods

### Data sources

A search was carried out for articles published in journals such as the Wanfangdata and Tsinghua-Tongfang using the keywords “upper arm circumference” to identify published or dissertations on UAC measurements. Hundreds of articles were considered, and ten of them included measurement values for UAC. The articles included participants in various regions of China, which involved Capital Beijing and central, northeast, southeast, northwest, and southwest provinces and cities in China. The UAC measurements in these articles were all the same to the pediatric measuring method generally used as the attachment of halfway point of the acromion to olecranon as a measurement point. Age groups in the studies varied (Table 1). Data were extracted and entered into an Excel database and were then analyzed with SPSS16.

**Table 1:** The 10 articles included in the pooled analysis

Order	Sitting	Areas of China	Data collection	Age(interval)	*N* (total)	*N* (boys)	*N* (girls)	Presentation	Method of measurements	Relative measuring
1	BJ	Capital Beijing	Cluster sampling in schools and kindergartens in urban and suburban	3–18 years (1 year)	19,705	10,151	9554	Dissertation 2009	Mid-upper arm circumference	Height, weight
2	SX(U,R)	Yangquan city of Shanxi province of central north	Hierarchy cluster sampling in 101 survey sets	0–5 years (0–6 months (1 months), 6–12 months (2 months), 1–2 years (3 months), 3–5 years (6 months))	6334	3262	3072	Pan 2011	Mid-upper arm circumference	Height, weight, circumference of chest and head
3	AH	Anhui province of central south	Random cluster sampling in 3 elementary schools and 2 junior high schools	6–17 years (2 years)	4476	2275	2201	Jiang 2002	Mid-upper arm circumference	Height, weight, circumference of chest, triceps skin-fold thickness
4	SZ	Suzhou city in Jiangsu province in Yangtze north	Cluster sampling in kindergartens	3–6 years (1 years)	1061	577	484	Liu 1988	Mid-upper arm circumference	Height, weight
5	ZJ	Zhejiang province in Yangtze south	Random sampling 1–3grade in 16 elementary schools	6–10 years (1 years)	1515	843	672	Tan 2004	Mid-upper arm circumference	Height, weight
6	IM	Hohhot city of Inner Mongolia of Northwest	Random sampling in 10 kindergartens	3–7 years (6 months)	3662	1936	1726	Wuyunrle 2000	Mid-upper arm circumference	Nine circumferences of chest, head, waist, and so on
7	LN	Dalian city of Liaoning province of Northeast	Random sampling	7–17 years (1 years)	1203	594	609	Xu 2000	Mid-upper arm circumference in Shao’s version	Four circumferences of chest, hip, minimum-waist, maximum-leg
8	GX(Miao)	Miao nationality in Guangxi municipality in Southwest	Miao nationality sampling	8–16 years (1 years)	814	454	360	Huang 2005	Mid-upper arm circumference in Wu Rukang’s version	Height, weight, skin-fold thickness, body fat
9	GX(Zhuang)	Zhuang nationality municipality of Guangxi of Southwest	Random sampling in 21 schools in 4 counties in 2 cities	7–18 years (1 years)	12,339	6825	5514	Tan 1994	Mid-upper arm circumference	Height, weight
10	GD	Guangzhou city of Guangdong province of Southeast	2 hierarchy cluster sampling in 10 kindergartens	4–8 years (1 years)	9108	4780	4328	Su 2003	Mid-upper arm circumference	Height, weight, skin-fold thickness

### Data processing

UAC data were summarized and weighted by age and gender. *Z* values were calculated and the mean and standard deviations were determined. Weighted smoothing of the curves was carried out after the means were connected. According to age and gender, the median average was *Z* = 0 ( *Z* > = −1 and *Z* < =1); *Z* = −1 (*Z* < −1 and *Z* > = − 2); *Z* = −2 (*Z* > −2 and *Z* < = −3); *Z* = −3 (*Z* > −3 and *Z* > = − 4); *Z* = 1 (*Z* > 1 and *Z* < = 2); *Z* = 2 (*Z* > 2 and *Z* < =3); and *Z* = 3 (*Z* > 3 and *Z* < =4). To calculate the mean and standard deviation with the *Z* value intervals, we obtained a smooth curve with exponential smoothing.

### Data analysis

The Chinese weighted values and the WHO data released online in 2006 (0- to 60-month-old children) were analyzed. Data, those compared and merged with growth curves by gender, were included. The *Z* values and exponential smoothing curve points were determined.

## Results

### Multicenter data distribution

Ten articles were included in the pooled analysis and participants from the northeast (DL), northwest (IM), southeast (GD), southwest (GX), the central plains (SX), central south (AH), and southwest (GD), and Yangtze areas (SZ and ZJ) of China, as well as Capital Beijing. The data on central Yangquan, Shanxi province, covered ages from 0 months to 4.5 years old and included both urban and rural children. Other multicenter reports based on a wide geographic distribution were representative of the general population and covered children who were 0~18 years old (Table 1) [7–15].

### Weighted comparison with the WHO study by gender

**Fig. 1:**
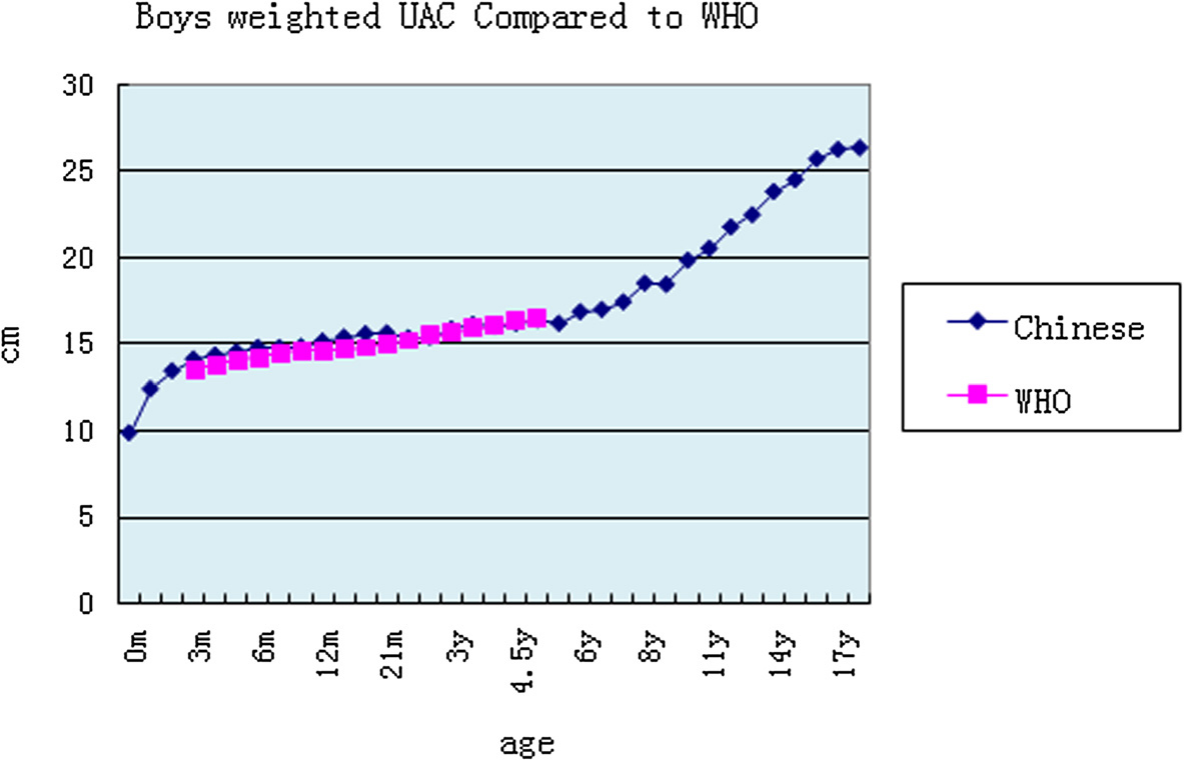
Boys weighted UAC compared to the WHO study

**Fig. 2:**
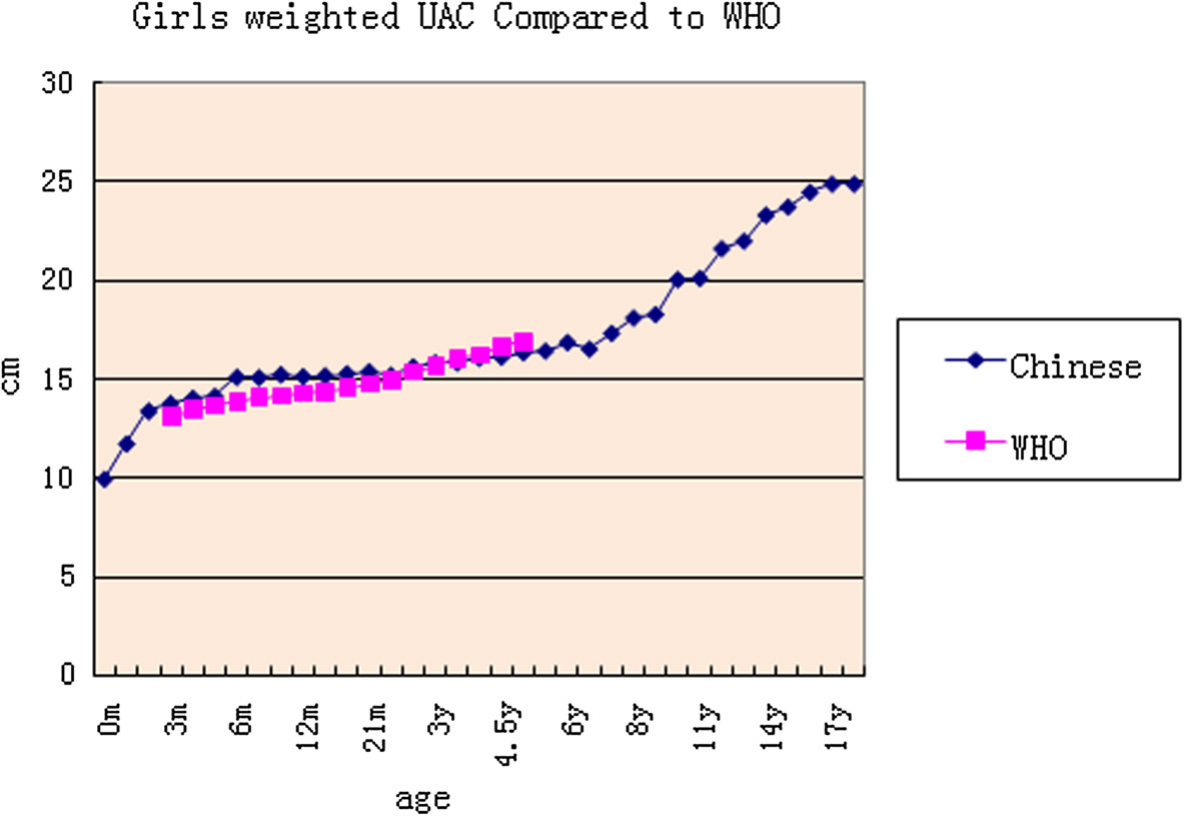
Girls weighted UAC compared to the WHO study

By gender, the weighted mean compared with the WHO study had a similar cross point at 3.5 years old. Differences with the WHO study were not obvious for boys and girls (Figs. 1 and 2).

### UAC weighted curves comparisons by gender

**Fig. 3:**
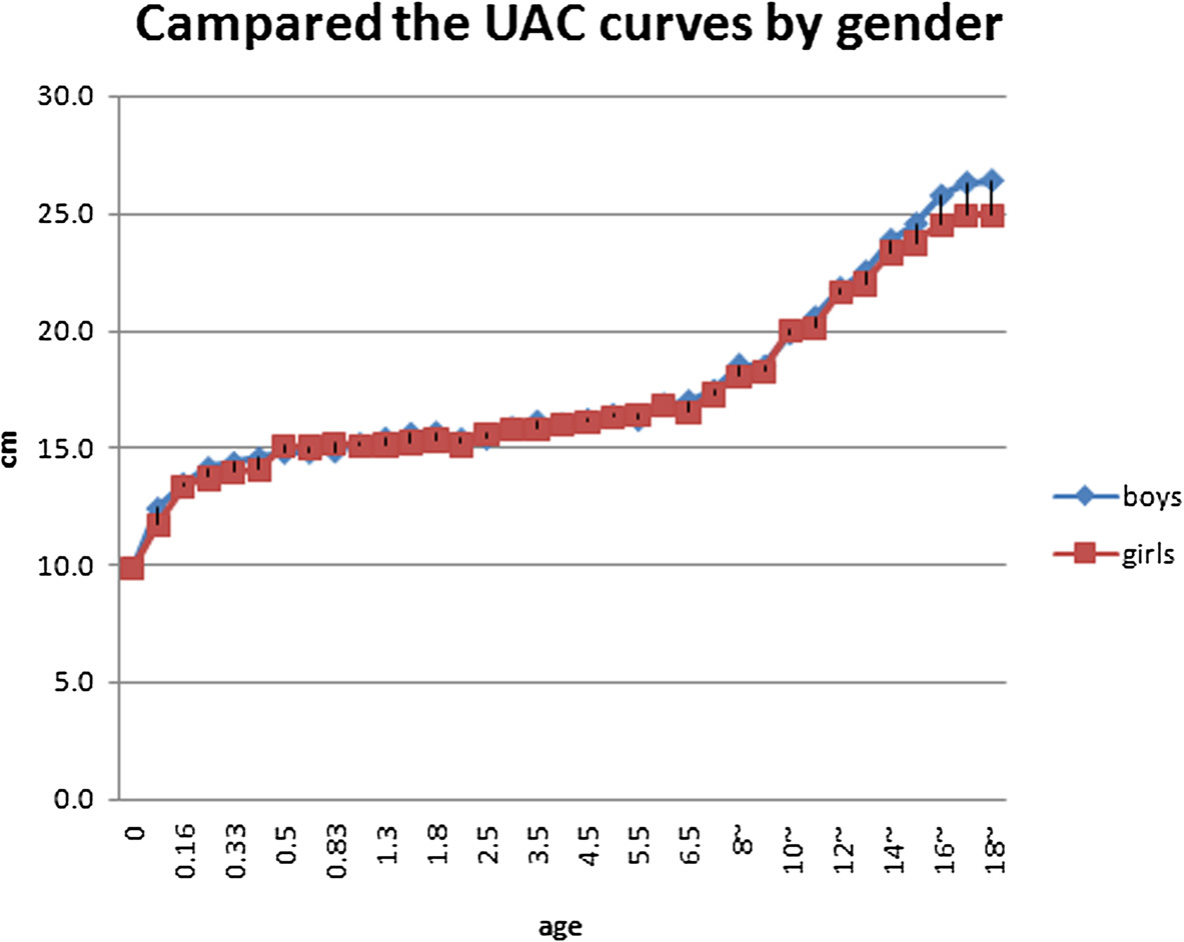
Compared UAC merged curves by Gender

**Fig. 4:**
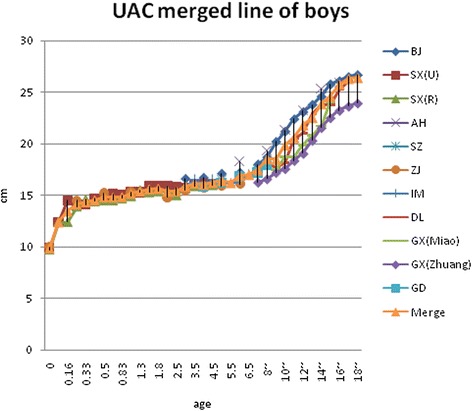
UAC curves comparing with boys

**Fig. 5:**
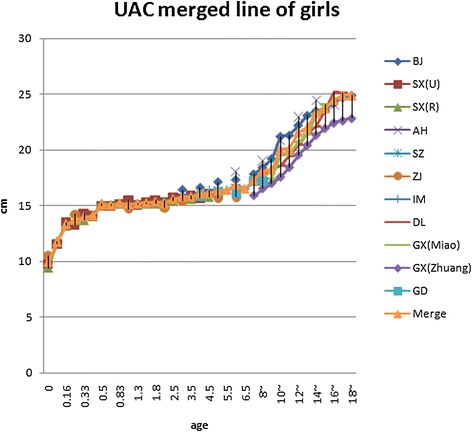
UAC curves comparing with girls

There were subtle differences in gender before puberty and more differences after puberty. The curve for boys was steeper (Fig. 3). Within each UAC curve chart, age groups cross-covered each other. The weighted curve integrated the studies smoothly, which had the possibility to add the *Z* score loci on it excluding 0–2 years (Figs. 4 and 5).

### Curve smoothing

**Fig. 6:**
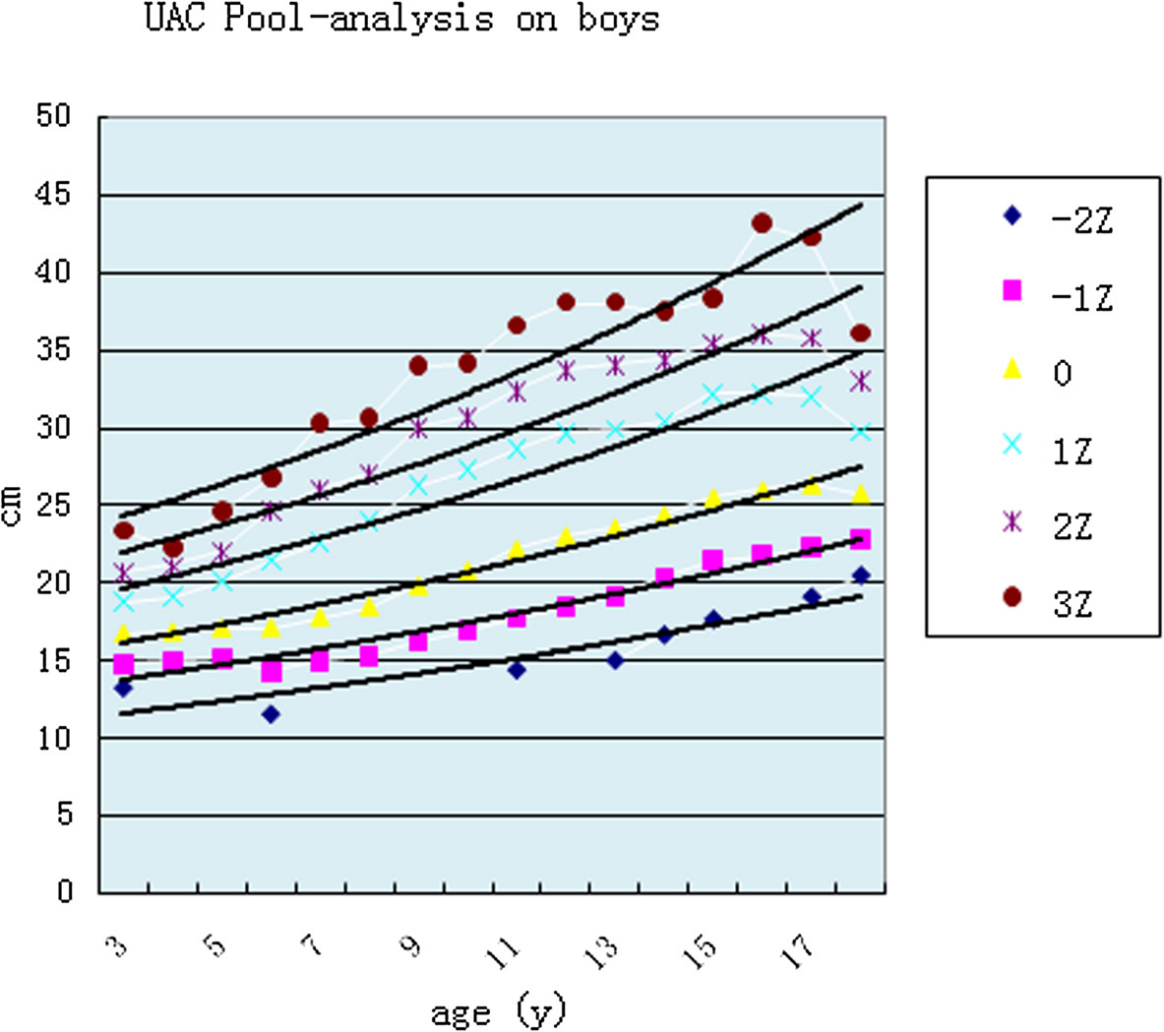
Pooled analysis for boys

**Fig. 7:**
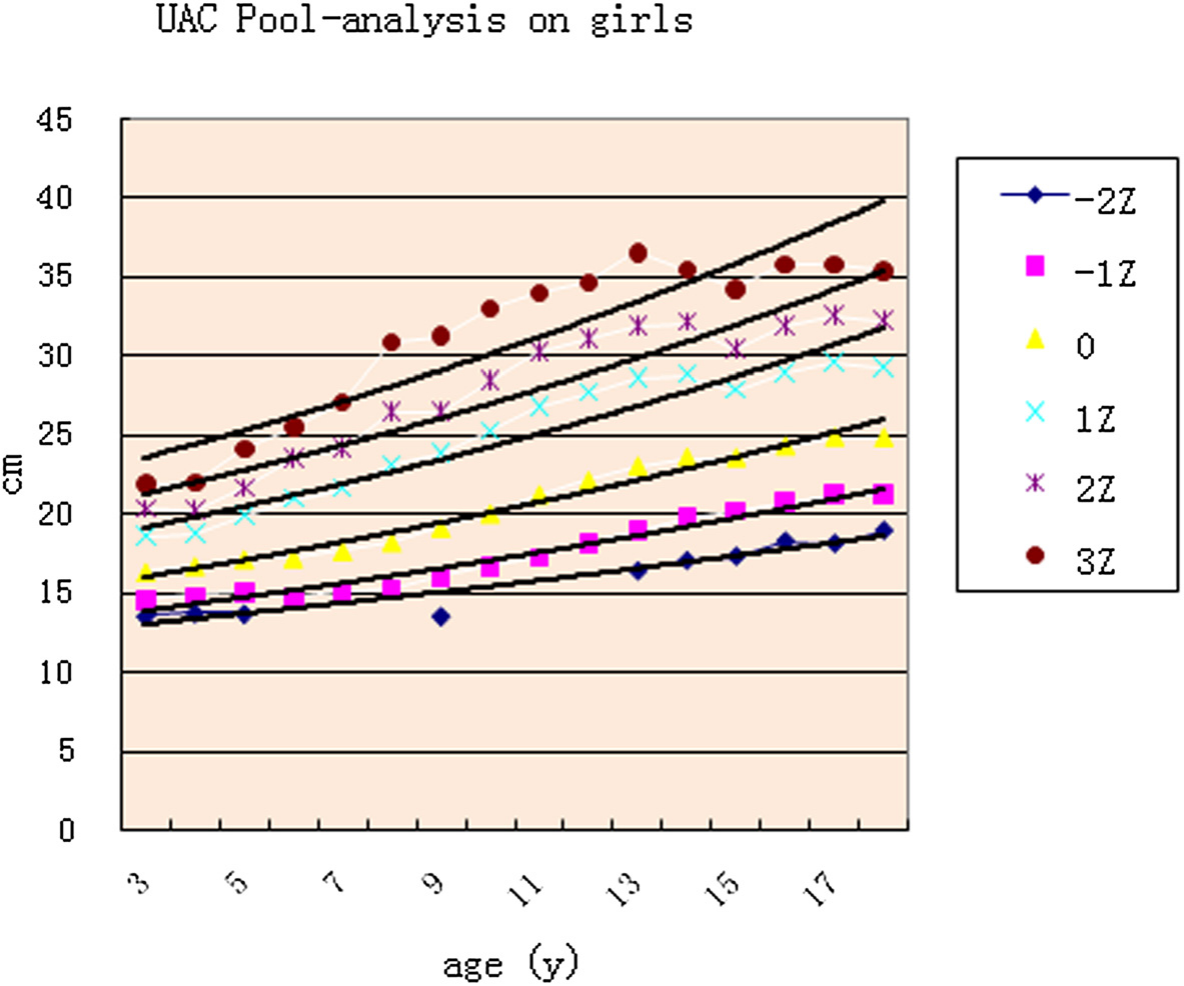
Pooled analysis for girls

The pooled analysis of the studies from the literature showed some regularities in *Z* scores for each locus, with boys and girls showing similar variation. After puberty, the values for boys were higher than for girls in the exponential smoothed curve (Table 2 and Figs. 6 and 7).

**Table 2:** The equations for Z score loci by gender

Girls	Boys
*Y*_0_ = 15.508*e*0.0321*X R* ^2^ = 0.9703	*Y*_0_ = 15.428*e*0.0361*X R* ^2^ = 0.9637
*Y*_+ 3_ = 22.786*e*0.0349*X R* ^2^ = 0.8277	*Y*_+ 3_ = 23.293*e*0.0402*X R* ^2^ = 0.8389
*Y*_+ 2_ = 20.447*e*0.0341*X R* ^2^ = 0.8883	*Y*_+ 2_ = 21.089*e*0.0384*X R* ^2^ = 0.8704
*Y*_+ 1_ = 18.491*e*0.0332*X R* ^2^ = 0.9263	*Y*_+ 1_ = 18.777*e*0.0383*X R* ^2^ = 0.8965
*Y*_− 1_ = 13.458*e*0.0294*X R* ^2^ = 0.9563	*Y*_− 1_ = 13.235*e*0.0337*X R* ^2^ = 0.8437
*Y*_− 2_ = 12.724*e*0.0235*X R* ^2^ = 0.9178	*Y*_− 2_ = 11.136*e*0.0337*X R* ^2^ = 0.8546

## Discussion

### Comparison with the WHO study

The age phases of the pooled analysis were broad; 0 to 60 months range was compared in the WHO study [6]. In our study, the weighted age boundary segmentation was the following: cutoff 1 month before a half year of age, 2 months after a half year old, 3 months at 2 years old, 6 months within 2–6 years of age, and 1 year during the range 7 to 18 years old. The comparison of results showed that there was some crossover with the curve of the WHO study but with the first segment showing values that a few above and then below the WHO study curve with a node located at 3.5 years. The reason may be that the curve of WHO study was obtained with exponential smoothing (http://www.who.int/nutrition/media_page). Many studies in the developing countries compared children’s UAC to WHO references as well. Reports have shown that a ROC curve can be used for evaluation of malnutrition in children under the age of five and UAC by age has better sensitivity than height in Kenya, Africa [16]. There were 26.8 % of children below 2 standard deviations from the mean in a study on children in Malaysia, Asia [17]. Nevertheless, some results implied that the use of the international reference may lead to lower specificity and predictive value in the screening of malnourished children in developing countries such as Uganda [18]. The average of children under 5 years old was at −2 standard deviations from the mean in a study carried out in Kenya [19]. Therefore, that seems to need a local UAC curve for references.

### Chinese children UAC curves integration

There were significant differences in adult UAC in China, because China is a large region and includes many nationalities. The Male Xinjiang Khalkhas, Tatar, and Xibo populations had a UAC of about 30 cm, which would be considered as brawnier. Ten southern ethnic groups, i.e., Hainan Han, Hui, Miao, DuLongZu, MuLaoZu, Buyi, Uighur, Tu, Mongolian, and Li had a UAC of about 24~25 cm, which would be considered as smaller. Women were divided into four types, and the strongest groups, in addition to the three of the male groups and ethnic Russians, had a UAC of about 28 cm. The Han, Uzbek, and Kazak urban and rural area populations in Jiangsu had a UAC of about 26 cm. Uely had a below average UAC of about 23~24 cm. The Hui, Soil, and Mongolian populations had a UAC of about 22~23 cm, which would be considered as thin [2–5, 20]. Asian children had less UAC than Caucasians [21]. In the present study, Children UAC development showed some differences in different regions, but the mean gaps between the values of highest and lowest curve of each age groups were all less than the standard deviation of their own study, although those were narrow before puberty and wider in adolescence (Figs. 4 and 5). The charts of the curves showed the merged curve was feasible made by combined studies, and the ligature of weighted mean UAC of age phases is also thought as the mean value of the reference curve. The *Z* score values could be added as well.

### Growth curve for each *Z* score

Many studies have supported the superiority of *Z* scores for UAC by age. Use of UAC with age- and sex- adjusted *Z* scores gave a better sensitivity than absolute cut of values in the identification of children wasting (acute undernutrition) [22–24]. Furthermore, regarding preschool obesity screening, the *Z* score of UAC by age seemed to be an appropriate alternative, which was better than UAC by height [25]. In addition, *Z* scores for other anthropometric indicators such as weight have showed symmetry if cut small in the right tail with *Z* score analysis [26]. UAC *Z* scores in this pooled analysis showed a normal distribution, similar to the curve for weight, which was a −2~+ 3 right normal curve. The index-smoothing trend showed some regularities with directions going up and down through various points in each Z trend. The development chart can be a representative reference for Chinese children and adolescents.

## Conclusion

The pooled analysis on UAC data for Chinese children and adolescents had a broad weighted value. The age group 0~60 months compared with the WHO curve showed there was some crossover with a little higher values for children younger than 3.5 years old and lower values for children older than 3.5 years old. *Z* scores ranged from −2 to ~3 and formed a normal distribution. Exponential smoothing was suitable for assessment.

The research has been approved by Ethics Committee of the Capital Institute of Pediatrics, within which the work was undertaken and that it conforms to the provisions of the Declaration of Helsinki.

## Abbreviations

UAC: mid-upper arm circumference
ROC: receiver operating characteristic curve
WHO: Would Health Organization
CDC: Center for Disease Control and Prevention
